# Infectious pseudo-aneurysm of the left ventricle: a case report and a review of the literature

**DOI:** 10.1186/s12872-019-01299-x

**Published:** 2020-03-24

**Authors:** Amadu E. Juliana, Kevin H. van ’t Kruys, Pieter G. Voigt, Nico A. Blom

**Affiliations:** 1 Department of Pediatric Cardiology, Leiden University Medical Center, Leiden, Netherlands; 2 Academic Pediatric Center Suriname, Academic Hospital Paramaribo, Paramaribo, Suriname; 3 Department of Cardio-thoracic Surgery, Academic Hospital Paramaribo, Paramaribo, Suriname

**Keywords:** Purulent pericarditis, Pseudo-aneurysm, Left ventricle, Children, Suriname

## Abstract

**Background:**

In the workup of a pediatric patient with pericarditis we found evidence of a pseudo-aneurysm of the left ventricle, which is a rare complication of purulent pericarditis.

**Case presentation:**

We present a case of a six-year-old girl who was diagnosed with pericarditis and a fistula between the pericardial and the intra-luminal space of the left ventricle of the heart. She was successfully treated with antibiotics and cardio-thoracic surgery. We found 23 published cases (21 with follow-up) of infectious pseudo-aneurysm of the heart, of which 19 underwent surgery, 5 had fatal outcome, and 2 who refused surgery survived. The majority of cases were associated with *Staphylococcus aureus*. The exact mechanisms of this rare complication remain unknown.

**Conclusions:**

A pseudo-aneurysm of the left ventricle is a rare and not well understood complication of a purulent pericarditis most commonly caused by *Staphylococcus aureus* infection. Because of risk of rupture, surgical intervention is advised.

## Background

Since the widespread use of antibiotics, purulent pericarditis became a relatively uncommon disease. In the workup of a pediatric patient with pericarditis we found evidence of a pseudo-aneurysm of the left ventricle, which is a rare complication of purulent pericarditis. We hereby describe our case and the available literature.

A literature search was performed on Medline using the following key words: pseudo-aneurysm, pericarditis, pancarditis, perimyocarditis. No filters were used to avoid missing relevant review articles and case reports. Also, the references of the relevant case reports and review articles were hand searched, to identify additional cases.

## Case presentation

**Fig. 1:**
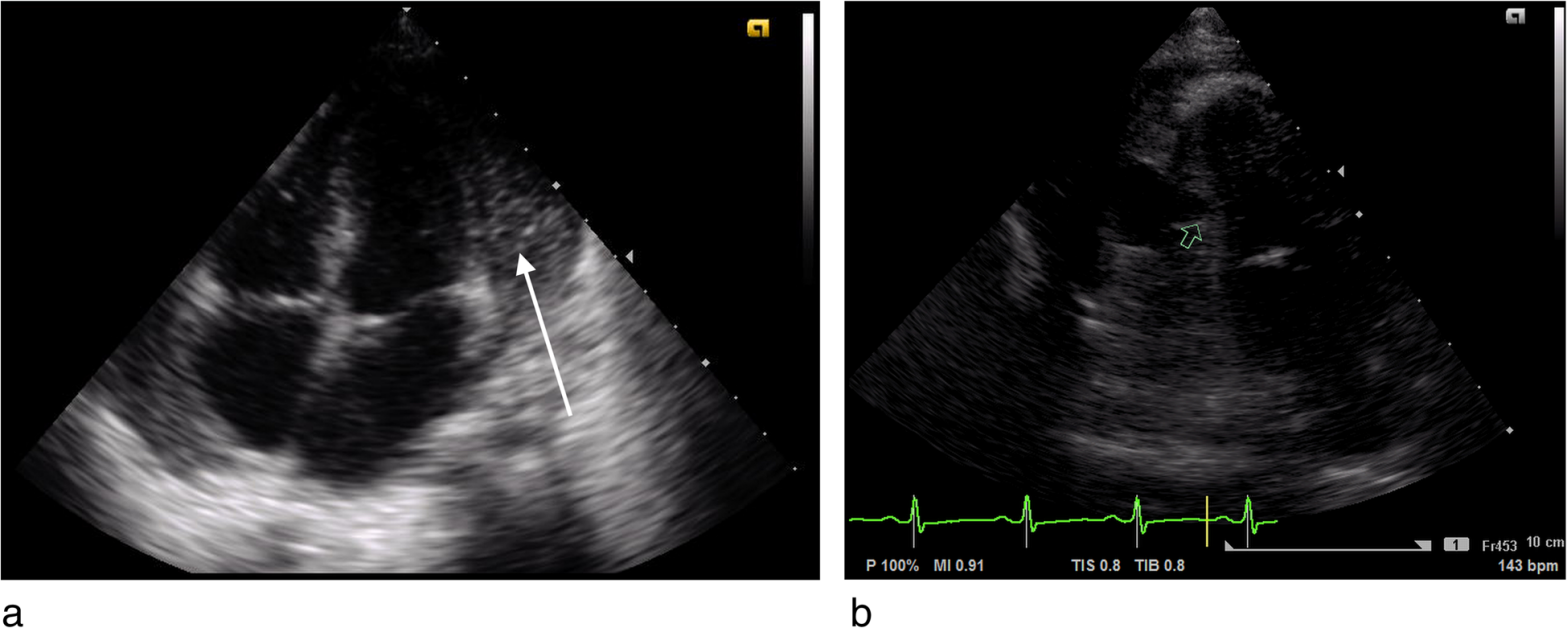
**a** Four chamber apical view, with organized purulent pericardial effusion 15–20 mm thick, with limited fluidity. **b** Apical 2 chamber view showing the left ventricle with an anechoic area (pseudo-aneurysm) lateral of the left ventricle free wall, with discontinuity of left ventricle wall, forming the communication between left ventricular inner space and the pericardial anechoic area


A previously healthy six-year-old girl was admitted with fever, dyspnea, abdominal pain, and pain in the right arm and shoulder. Four days before admission she had fallen from a tree while playing. Shortly after admission she was transferred to the ICU because she acutely developed signs of shock, anemia and progressive abdominal pain. Splenic rupture was suspected and confirmed by ultrasound showing sub-capsular spleen hematoma and free intra-abdominal fluid. During the ICU stay she developed an abscess on the right shoulder, for which antibiotic treatment was started. The culture of the aspirate, taken before start of antibiotics, from this abscess was positive for *Staphylococcus aureus*. After 1 month she was transferred to our center under the suspicion of pericarditis, because of persistent fever and cardiomegaly on the chest X-ray. Echocardiography revealed pericardial effusion, located mostly behind the left ventricular (LV) posterior wall (Additional file 1), with a to and fro blood flow through a fistula between the left ventricular lumen and a cavity in the pericardial space, and echogenic densities suggestive for fibrin strands and clots (see Fig. 1). Cardiac function was good with mild mitral valve regurgitation. Findings were suggestive for advanced purulent bacterial pericarditis complicated by covered left ventricular perforation (pseudo-aneurysm).

**Additional file 1: ** Colour Doppler showing blood shunting to and fro between left ventricle and the anechoic intra-pericardial area (pseudoaneurysm).


High doses of intravenous cefotaxime and flucloxacillin, already started in the referring hospital, were continued. She developed arthritis of the left knee and right elbow which were both aspirated but with negative cultures. Two weeks after admission her clinical condition did not improve with persistent leukocytosis and high C-reactive protein levels. A pericardial abscess with ongoing bacteremia was suspected and surgical intervention was scheduled.

**Fig. 2:**
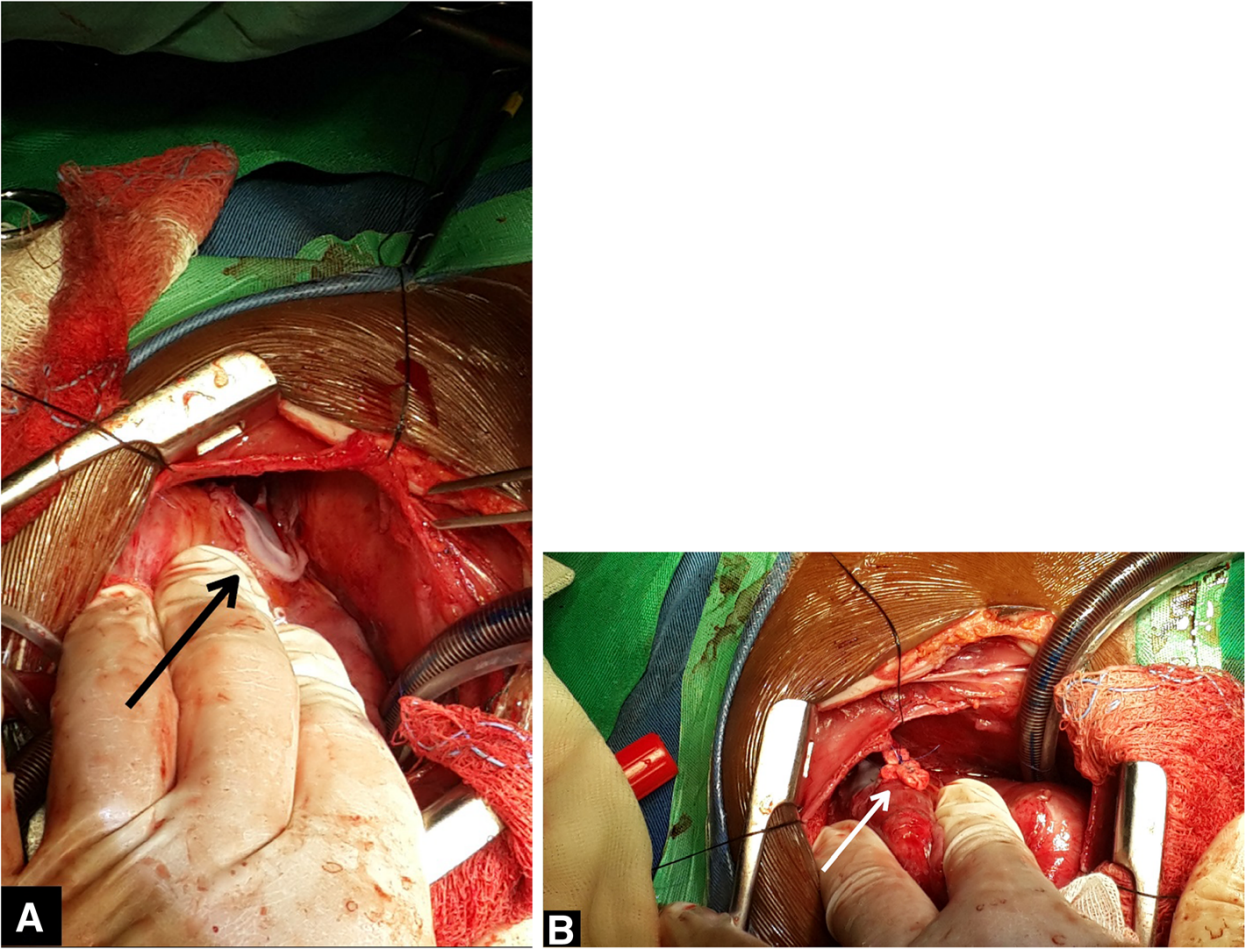
**a** the pseudo-aneurysm visible (black arrow) on the posterior-lateral wall of the left ventricle. **b** the pseudo-aneurysm closed with proline sutures with felt (white arrow)

A median sternotomy was performed, leaving the pleural space closed. The patient was placed on extracorporeal circulation using standard bicaval cannulation. The operation was performed on a beating heart. On opening of the pericardium multiple small abscesses and adhesions were identified and removed, as were thick vegetations on the left ventricular posterior wall. The fistula in the LV myocardium connecting the LV lumen with an abscess on the posterior-lateral wall of the LV was found and closed with prolene sutures with felt (see Fig. 2). The pericardial space was irrigated multiple times with a solution of sodium-chloride and iodine. Cultures of the abscesses were negative. Post-operatively she had a quick recovery without fever episodes. She was discharged from the hospital in good condition 2 weeks after surgery. Antibiotics were continued for almost 4 months because of persistent osteomyelitis of the right upper arm and a septic arthritis of the right elbow. Echocardiograms during follow-up revealed no abnormalities.

## Discussion and conclusion

We report a pediatric case of left ventricle pseudo-aneurysm as a complication of purulent pericarditis secondary to a *Staphylococcus aureus* osteomyelitis. The pericarditis and pseudo-aneurysm were effectively treated with antibiotics, surgical drainage of the pericardium and closure of the fistula.

A left ventricular pseudo-aneurysm is a rare complication of a bacterial pericarditis. Our literature search revealed a total of 23 cases, including 12 children, with outcome reported in 21 cases (see Table 1). In 1981 Sadan et al. first reported a left ventricle pseudo-aneurysm as a complication of a purulent pericarditis in a 6-year-old girl [1]. *Staphylococcus aureus* was the most common causative microorganism occurring in 52% of cases, excluding MRSA. Mycobacterium Tuberculosis was reported in three patients. The most common location of the pseudo-aneurysm was at the posterior and/or lateral wall of the left ventricle. Echocardiography was the most frequently used imaging tool to obtain diagnosis. Nineteen patients underwent an operation because of the risk of rupture or ongoing infection. Four patients died post-operatively, including one 18-year-old male and three adult patients. The causes of death in these four cases were profuse bleeding because of a serious coagulopathy postoperatively, further deterioration because of the widespread infection despite surgery, persistent hypotension postoperatively and the inability to repair the LV pseudo-aneurysm (LVPA) with eventually rupturing of the LVPA. The outcomes in the other cases that underwent surgery were uneventful and all these patients recovered fully. It is intriguing that in the two of the three reported cases of patients that refused surgical treatment no rupture occurred during follow-up [8, 12]. One case was a 79-year-old woman who had a pseudo-aneurysm that initially increased in size but remained stable after 1 year of follow up [12]. The other case was a 13-year-old child with a pseudo-aneurysm that remained stable. Echocardiographic examination after more than 6 years revealed moderate echogenicity adjacent to the lateral wall of the left ventricle without discontinuity of the left ventricular wall [8]. From the third case, a 3-year-old female child, there was no further information available because of lost to follow-up [21]. In theory the risk of spontaneous rupture is higher in pseudo-aneurysms compared to true aneurysms because of the lack of a muscle layer.

**Table 1:** Overview of published case reports of a left ventricular pseudo aneurysm after purulent peri/pancarditis

Study (Year of publication), [ref]	Age (years)	Sex	Location pseudoaneurysm	Diagnostic methods	Micro-organism	initial presentation	Outcome
Sadan et al. (1981) [1]	6	F	Left ventricular posterior wall	Left ventriculogram	Staph. Aureus	Femoral osteomyelitis	Survived
Wiegers et al. (1988) [2]	28	F	Left ventricular postero-medial wall	Echocardiogram Left ventriculography	Staph. Aureus	Intravenous drug abuser/bacteremia	Died
Roberts et al. (1991) [3]	33	F	Left ventricular posterior wall	Magnetic Resonance Imaging Echocardiogram Left ventriculography	Staph. Aureus	Intravenous drug abuser/pericarditis	Not reported
Campos et al. (1996) [4]	8	M	Left ventricular posterior wall	Echocardiogram Computed Tomographic Scanning Magnetic Resonance Imaging	Staph. Aureus	Femoral osteomyelitis	Survived
de Boer et al. (1999) [5]	2	F	Left ventricular wall	Echocardiogram	Staph. Aureus	Chicken pox/bacteremia	Survived
Moraes et al. (1999) [6]	4	F	Left ventricular posterolateral wall	Echocardiogram Left ventriculogram	Staph. Aureus	Pneumonia and subsequent septic arthritis	Survived
Osula et al. (2001) [7]	33	F	Left ventricular free wall (apical)	Echocardiogram Left ventricular angiogram	Staph. Aureus	Intravenous drug abuser/bacteremia	Survived
Deng et al. (2003) [8]	13	F	Left ventricular lateral wall	Echocardiogram	Staph. Aureus	Pneumonia	Survived
Yamabi et al. (2004) [9]	35	M	Left ventricular posterior wall	Echocardiogram Left ventricular angiogram	Staph. Aureus	Sepsis	Survived
Arifi et al. (2004) [10]	20	F	Left ventricular wall	Echocardiogram Computed Tomographic Scanning	Staph. Aureus	Sepsis/endocarditis mitral valve	Survived
Vijayvergiya et al. (2008) [11]	52	M	Left ventricular anterolateral wall	Echocardiogram Computed Tomography Angiography	*Streptococcus viridans*	Pyopericardium/empyema	Died
Chen et al. (2010) [12]	79	F	Posterior wall of the left ventricle	Computed Tomography Echocardiogram	Staph. Aureus	Bacteremia/pericarditis	Survived
Wei et al. (2014) [13]	18	M	Left ventricular posterolateral wall	Echocardiogram Computed Tomographic Scanning	*Mycobacterium tuberculosis*	Dyspnea low grade fever.	Died
Nair et al. (2014) [14]	6	M	Left ventricular posterolateral wall	Echocardiogram Computed Tomography Angiography	MRSA Acinetobacter species	Sepsis	Survived
Desai et al. (2015) [15]	2	F	Left ventricular lateral wall	Echocardiogram Computed Tomography Angiography	No growth of organisms	Intermittent fever	Survived
Krishna et al. (2015) [16]	1.5	F	Left ventricle below posterior mitral leaflet	Trans esophageal echocardiography	Staph. Aureus	Fever/pericarditis	Survived
Lindblom et al. (2015) [17]	48	M	Left ventricular posteroinferior wall	Echocardiogram Computed Tomography	No growth of organisms	Fever/pericarditis	Survived
Sunkara et al. (2015) [18]	48	M	Left ventricular basal infero-lateral wall	Echocardiogram Computed Tomography Angiography	Streptococcus Viridans	Pericarditis	Died
Arora et al. (2015) [19]	6	M	Left ventricular inferior septal wall	Echocardiogram	MRSA	Sepsis/arthritis	Survived
Yeh et al. (2016) [20]	46	M	Left ventricular antero-lateral wall	Computed Tomography Cardiac catheterization	*Klebsiella pneumoniae*	Fever/Pericarditis	Survived
Sreevathsa et al. (2018) [21]	9	M	Left ventricular posteroinferior wall	Echocardiogram Computed Tomography Left ventricular angiography	Mycobacterium Tuberculosis	Retrosternal chest pain/palpitations	Survived
Sreevathsa et al. (2018) [21]	3	F	Posterior wall of the left ventricle	Not reported	Possible Mycobacterium Tuberculosis	Tibial osteomyelitis	Not reported
Sreevathsa et al. (2018) [21]	6	F	Left ventricular posterolateral wall	Echocardiogram	Possible Varicella Zoster	Varicella zoster infection	Died

*F* Female, *M* Male, *Staph. Aureus Staphylococcus Aureus*, *MRSA* Multi resistant *Staphylococcus Aureus*

Purulent pericarditis is a rare disease commonly caused by gram-positive cocci, among which *Staphylococcus aureus* is the most commonly detected micro-organism. Because of its virulence and ability to destroy or disrupt cardiac structures, complications may occur rapidly after onset of infection [6]. Infection of the pericardium occurs from either direct or hematogenous seeding of organisms from the primary source. There are several hypotheses regarding the development of pseudo-aneurysms as a complication of purulent pericarditis. It may be due to a direct spread of infection from pericardium (most probable mechanism in our case), and or an intra-myocardial abscess that gradually perforates the ventricular wall and contained within the thickened pericardium or fibrous tissue. Others have suggested a possible role of the enzyme staphylokinase which converts plasminogen to plasmin, which stimulates collagen breakdown [1, 11, 15, 22]. Why the pseudo-aneurysm is located most commonly in the posterolateral (submitral) region remains unanswered. One author stated that because hospitalized patients usually are in the recumbent position, an inflammatory reaction of the posterior pericardium may result in pericardial adhesions and the formation of a posterior left ventricular pseudo-aneurysm rather than cardiac tamponade [23].

In the present case purulent pericarditis secondary to bacterial osteomyelitis is most probably the cause of the ventricular pseudo-aneurysm. Since there was growth of *Staphylococcus aureus* from the aspirate of the abscess from the right shoulder, purulent pericarditis is likely the result of hematogenous seeding. Cultures from the operative specimens remained negative which is most likely explained by the extensive antibiotic treatment prior to surgery. In line with most other reported cases we decided that surgical treatment was needed because of ongoing infection and high risk of rupture. Peri-operatively the intra-cardiac abscess was found at the posterior lateral wall of the left ventricle, a finding consistent with most of the cases discussed. The exact mechanisms causing post-pericarditis pseudo-aneurysm and the tendency for Left posterolateral location remain unknown.

Since we have no knowledge of unpublished cases (with or without surgery), it is likely that there is publication bias, which asks for caution in interpreting the overall outcome.

In summary, this is a case of a 6-year-old girl with pseudo-aneurysm of the left ventricle as a complication of purulent pericarditis secondary to osteomyelitis, with good recovery after antibiotics and cardiac surgery. A pseudo-aneurysm of the left ventricle is a rare and not well understood complication of a purulent pericarditis most commonly caused by *Staphylococcus aureus* infection. Because of risk of rupture, surgical intervention is advised, although there are two reported cases that survived without surgery.

## Data Availability

All data that was generated or analyzed during the current study are available from the corresponding author on reasonable request.

## Abbreviations

ICU: Intensive care unit
LV: Left ventricular
LVPA: Left ventricular pseudo-aneurysm
MRSA: Multi resistant *Staphylococcus Aureus*
